# Genome-wide identification of the *N*^6^-methyladenosine regulatory genes reveals *NtFIP37B* increases drought resistance of tobacco (*Nicotiana tabacum* L.)

**DOI:** 10.1186/s12870-024-04813-2

**Published:** 2024-02-26

**Authors:** Huan Su, Lijun Meng, Zechao Qu, Wei Zhang, Nan Liu, Peijian Cao, Jingjing Jin

**Affiliations:** 1 Beijing Life Science Academy, Beijing, 102200 China; 2 China Tobacco Gene Research Center, Zhengzhou Tobacco Research Institute of CNTC, Zhengzhou, 450001 China; 3 China National Tobacco Quality Supervision & Test Center, Zhengzhou, 450003 China

**Keywords:** RNA methylation, m^6^A regulators, Tobacco, Expression pattern, Drought stress

## Abstract

**Background:**

*N*^6^-methyladenosine (m^6^A) is one of the common internal RNA modifications found in eukaryotes. The m^6^A modification can regulate various biological processes in organisms through the modulation of alternative splicing, alternative polyadenylation, folding, translation, localization, transport, and decay of multiple types of RNA, without altering the nucleotide sequence. The three components involved in m^6^A modification, namely writer, eraser, and reader, mediate the abundance of RNA m^6^A modification through complex collaborative actions. Currently, research on m^6^A regulatory genes in plants is still in its infancy.

**Results:**

In this study, we identified 52 candidate m^6^A regulatory genes in common tobacco (*Nicotiana tabacum* L*.*). Gene structure, conserved domains, and motif analysis showed structural and functional diversity among different subgroups of tobacco m^6^A regulatory genes. The amplification of m^6^A regulatory genes were mainly driven by polyploidization and dispersed duplication, and duplicated genes evolved through purified selection. Based on the potential regulatory network and expression pattern analysis of m^6^A regulatory genes, a significant number of m^6^A regulatory genes might play important roles in growth, development, and stress response processes. Furthermore, we have confirmed the critical role of *NtFIP37B*, an m^6^A writer gene in tobacco, in enhancing drought resistance.

**Conclusions:**

This study provides useful information for better understanding the evolution of m^6^A regulatory genes and the role of m^6^A modification in tobacco stress response, and lays the foundation for further elucidating the function of m^6^A regulatory genes in tobacco.

**Supplementary Information:**

The online version contains supplementary material available at 10.1186/s12870-024-04813-2.

## Background

Epigenetic modifications are mechanisms that affect gene expression and function directly without altering biological gene sequences that can be passed on to offspring. Nowadays, epigenetic regulations have been found to involve in DNA/RNA methylation, histone modification, chromatin remodeling, and noncoding RNA profile changes [1, 2]. DNA methylation and histone acetylation are important strategies for the fine regulation of developmental processes and the response to environmental stresses. RNA modification, which is analogous to DNA methylation in function, also plays a prominent role throughout the life cycle of organisms. Initially discovered in mammals, chemical modifications of RNA are subsequently found to be prevalent in all living things [3]. More than 160 types of RNA modifications have been identified, most of which are present in transfer RNA (tRNA) and ribosomal RNA (rRNA). Methylation at the sixth *N* of adenosine, also known as *N*^6^-methyladenosine (m^6^A), is the earliest and most common type of mRNA modification. Further research has revealed that m^6^A modification also exists in non-coding RNAs, such as circular RNA (circRNA) [4] and long non-coding RNA (lncRNA) [5]. In eukaryotes, m^6^A modification is responsible for about 80% of all RNA methylation modifications. The identification of the m^6^A demethylase FTO (Fat Mass and Obesity-Associated Protein) [6] and its homologue *ALKBH5* (α-ketoglutarate-dependent dioxygenase) in mammals has confirmed the dynamic nature of m^6^A modification [7].

The degree of m^6^A modification is governed by two classes of proteins, known as methyltransferase (m^6^A writer) and demethylase (m^6^A eraser), which add or remove methyl groups to the adenine located in the RNAs’ conserved motif. To recognize m^6^A methylation on RNA base sites, specific reading enzymes (m^6^A reader) are also needed. Reader proteins recognize the m^6^A methylation motifs and bind specifically, thereby regulating downstream translation, mRNA nucleation transport speed, and mRNA degradation [8]. In summary, the precise regulatory network formed by writers, erasers, and readers governs the production, clearance and functional realization of m^6^A modification in biological organisms.

The m^6^A modification is catalyzed by a highly conserved multi-component methyltransferase complex (MTC, also known as the m6A writer complex). In mammals, MTC comprises of two sub-complexes, designated as m^6^A methyltransferase-like (METTL) complex (MAC) and m^6^A METTL-associated complex (MACOM) [1, 9]. MAC composes of the methylase heterodimer of methyltransferase-like 3 (METTL3) and METTL14, which comprises the catalytic core of the m^6^A methyltransferase [10, 11]. MACOM includes Wilms’ tumor 1 associated protein (WTAP), VIRMA (KIAA1429), HAKAI, RNA Binding Motif Protein 15 (RBM15), and its paralog RBM15B, as well as the recently discovered zinc-finger CCCH domain-containing protein 13 (ZC3H13) [12–16]. Similar with the mammalian counterparts, the m^6^A methyltransferase complex has been systematically identified in *Arabidopsis*, demonstrating its two core methyltransferases MTA (ortholog of METTL3) and MTB (ortholog of METTL14), and several accessory proteins FKBP12 INTERACTING PROTEIN 37 KD (FIP37, ortholog of WTAP), VIRLIZER (VIR, ortholog of VIRMA/KIAA1229), HAKAI [17]. In contrast, another known methyltransferase FIONA1 (ortholog of METTL16) functions separately for the deposition of m^6^A markers on mRNA [18, 19]. In *Arabidopsis*, recent research has proposed HIZ2, a HAKAI-interacting zinc finger protein, as a plausible plant analogue to ZC3H13, but the precise biological role of HIZ2 within the m^6^A writer complex is still undetermined [20].

Dynamic modifications of m^6^A have been recognized as a crucial regulator of plant growth, development, and stress response, as they influence the expression of corresponding genes through methylation modification. M^6^A modification in plants performs three main functions: mRNA processing, plant growth and development, and stress response [21]. Typically, the regulation of RNA metabolism of a single transcript is associated with the position of m^6^A marks. Specifically, m^6^A modification at the 3' UTR and stop codon sites primarily governs transcript stability and transcriptome integrity, whereas m^6^A modification in the 5' UTR assists in translation regulation and those in the coding region affect mRNA stability and splicing [17, 21, 22]. In *Arabidopsis*, m^6^A modification in the 3' UTR and 5' UTR is positively related to gene expression, while modification in other regions leads to reduced gene expression [23]. Research suggests that m^6^A modification plays a significant role in the embryonic development of plants. Reduction in the post-embryonic expression level of m^6^A methyltransferase, including *MTA*, *MTB*, *FIP37*, *VIRILIZER*, and *HAKAI*, caused a sharp decline in m^6^A. Knocking out the genes that encode core writer genes results in embryonic death [24–26]. Additionally, various developmental processes, such as flowering transition [19, 27], trichome and leaf morphology [28] and fruit maturation [29] are also influenced by m^6^A modification. In response to abiotic stresses, such as drought [30, 31], salt [32], cold [33], and biotic stresses, including bacterial infection [34], m^6^A can either promote or inhibit the expression of related genes. However, the current research on m^6^A is limited to a few model plants, highlighting the need for further research in other plants.

Common tobacco (*Nicotiana tabacum* L.) is a widely cultivated non-food crop, which is also commonly utilized as a model plant for genetic research. Furthermore, as a classical allotetraploid species, it plays a critical function in evolutionary studies. Recent studies have demonstrated that the infection of tobacco mosaic virus (TMV) has altered the level of m^6^A within tobacco [35]. Additionally, overexpression m^6^A methyltransferase ClMTB of watermelon enhanced the drought tolerance of tobacco [36]. However, genes associated with m^6^A modification in tobacco have yet to be identified. In this context, we conducted a comprehensive analysis of the classification, evolution, gene structure, and potential interaction network of m^6^A regulatory genes in tobacco, as well as studied the patterns of gene expression at different stages of tissue development and in response to a range of biotic and abiotic stresses. In addition, we validated the significant roles of *NtFIP37B* in drought resistance of tobacco. This study provides profound insights into the biological functions of m^6^A regulatory genes in tobacco and reveals the potential m^6^A-mediated regulatory mechanism in stress response.

## Materials and methods

### Identification and characterization of m^6^A regulatory genes in tobacco

To identify all members of m^6^A regulatory genes in tobacco, the protein sequence of m^6^A regulatory genes in *Arabidopsis*, tomato and rice were obtained from The Arabidopsis Information Resource (TAIR) [37], Sol Genomics Network (SGN) [38] and Ensembl Genome database [39], respectively. These sequences were used as queries against the tobacco genome [40] using BLAST program with the parameter E-value < 10^–5^. Then, HMMER v3 [41] was applied to identify candidate of m^6^A regulatory genes using hidden Markov models (HMM) based on m^6^A conserved domains: the MT-A70 family (PF05063), WTAP family (PF17098), VIRILIZER domain (PF15912), AdoMet_MTases domain (PF05971), 20G-Fe (II) oxygenase superfamily (PF14532) and YTH domain (PF04146) [42]. Candidates with incomplete domains were further removed by Conserved Domain Search (CD-Search) [43]. The physicochemical parameters of the m^6^A regulatory proteins, including molecular weight (MV), and isoelectric points (PI), were examined using TBtools [44]. Protein subcellular localization was predicted by WoLF PSORT Online software (https://wolfpsort.hgc.jp/) [45].

### The phylogenetic classification, gene structures and conserved motifs analysis

An unrooted phylogenetic tree was generated by MEGA 7 [46] using the neighbor-joining method with bootstrap value of 1,500. ITOL (https://itol.embl.de/) [47] was used to visualize phylogenetic trees. The protein sequences of dicotyledonous species (*Arabidopsis thaliana*, *N. tabacum*, *Solanum lycopersicum*, *Vitis vinifera*, and *Gossypium hirsutum*), monocotyledonous species (*Zea mays*, *Triticum aestivum*, and *Oryza sativa*), pteridophyte species (*Selaginella moellendorffii*), moss species (*Marchantia polymorpha* and *Physcomitrella patens*), and gymnospermous species (*Pinus tabuliformis*) for the evolutionary tree construction were derived from previous studies [48, 49]. TBtools software [44] was used to visualize the structures of m^6^A regulatory genes in tobacco. MEME software [50] was employed to analyze the conserved motifs in m^6^A regulatory proteins with maximum number of motifs of 10. InterProScan [51] was used to annotate the identified motifs.

### The chromosomal distributions, gene duplication and collinearity analysis

All tobacco m^6^A regulatory genes were mapped to their respective chromosomes. To infer the origin of m^6^A regulatory genes in allotetraploid tobacco, m^6^A regulatory genes of its two ancestors of the modern diploids: *Nicotiana sylvestris* and *Nicotiana tomentosiformis* were identified by the same method as above. Then, the origin, loss and expansion of m^6^A regulatory gene in tobacco were inferred through phylogenetic tree. DupGen_finder [52] program was used to explore the duplication events of m^6^A regulatory genes in tobacco, with *Arabidopsis* as the outgroup. For these duplicated gene pairs, KaKs_Calculator3.0 [53] was used to calculate the non-synonymous (*Ka*) and synonymous substitution (*Ks*) rates. The protein sequences of duplicated m^6^A regulatory gene pairs were used to calculate the divergence time with the formula $T={K}_{s}/(2 \times 1.5\times {10}^{-8})\times {10}^{-6}$ [54]*.* TBtools [44] and MCScanX [55] were used for the collinearity analysis of m^6^A regulatory genes among different species.

### Promoter analysis and interaction network prediction

The STRING v11.5 database [56] was used to construct the protein–protein interaction network among m^6^A regulatory genes in tobacco. The 2,000 bp upstream sequences from the transcription start site, were collected and submitted to the PlantCare [57] database for *cis*-acting element analysis. Mature miRNAs was downloaded from the miRBase database [58]. The psRNATtarget database [59] was used to search the regulatory relationship between miRNAs and tobacco m^6^A regulatory genes, with an Expectation threshold of < 5 and other parameters at their default values. Cytoscape [60] was used to visualize the interaction network.

### Expression profiles based on transcriptome data

Transcriptome data from eight representative tobacco tissues (root, stem, young leaf, mature leaf, senescent leaf, immature flower, mature flower and senescent flower) were utilized to investigate tissue-specific expression patterns of m^6^A regulatory genes. Additionally, tobacco transcriptome samples subjected to various biotic stresses such as Potato Virus Y (PVY), black shank (BS), Cucumber mosaic virus (CMV), and *Ralstonia solanacearum* (RS), as well as abiotic stresses including high/low temperature, drought, salt, cadmium, and topping, were obtained from the NCBI SRA database to investigate the response of m^6^A regulatory genes to different stresses. The accession numbers for all the transcriptome samples utilized in this study could be found in Table S2. The Salmon software (v1.10.1) [61] was employed to perform quantification on all clean reads, while the R package ‘tximport’ (https://github.com/mikelove/tximport) was utilized for calculating the transcripts per kilobase million (TPM) values. Visualization of expression profiling was performed by TBtools software.

### 3D structure predictions and protein docking

The three-dimensional (3D) structures of m^6^A regulatory proteins were predicted using the homology modeling method. All m^6^A regulatory proteins were queried against the Protein Data Bank (PDB) to identify the best template, and the models were built by Swiss-Model [62]. The protein docking among tobacco m^6^A writer proteins were performed using HDOCK software [63], and the model with the lowest docking energy score, which represents the best quality of docking, was retained for subsequent analysis. PyMOL software (https://pymol.org/2/) [64] was used to visualize these models.

### Plant materials and stress treatments

The tobacco cultivar K326 was employed to investigate the tissue-specific and stress-induced expression patterns of m^6^A regulatory genes in tobacco. Briefly, the seedlings were grown in plastic pots with a 16-h light cycle at 28°C during the day and 23°C at night. Similar with our previous study [65], root, stem, leaf, bud tissues at seedling stage (SS), vigorous growth stage (VG), and flowering stage (FS), as well as petal, receptacle, stamen, and pistil samples of reproductive organs (RO), were collected from tobacco. The drought and cold stress were conducted during the vigorous growth stage of tobacco. For drought treatment, 28-day-old tobacco plants were subjected to 7 days of water withholding. Cold treatment was conducted in a growth chamber with the temperature set at 4°C. Untreated plantlets were used as controls. After treatment, the treated and control plantlets were collected and immediately frozen in liquid nitrogen, then stored at -80°C for further experiments. All samples were independently conducted with three biological replicates.

### RNA isolation and relative expression analysis

Total RNA was extracted from the above-mentioned tobacco samples using the SuperPure Plantpoly RNA Kit (Gene Answer, Beijing, China). RNase-free DNase I (Gene Answer) was used to eliminate the DNA contamination. First-strand cDNA synthesis was performed using 1 μg of total RNA as a template using reverse transcriptase M-MLV (Takara Biomedical Technology, Beijing, China), and the resulting cDNA was diluted to a concentration of 50 ng/µL. The quantitative real-time polymerase chain reaction (qRT-PCR) of tobacco m^6^A regulatory genes was performed using a 20 μL reaction system and employing the SYBR Green kit (Imagene, Beijing, China). The PCR program included an initial step of 95°C for 30 s, followed by 40 cycles of 95°C for 10 s, and 60°C for 30 s. The relative gene expression was calculated using 2^−∆∆Ct^ method, and the expression levels were standardized to the expression level of the *GAPDH* gene. All specific primers for qRT-PCR are listed in Table S3. Three independent biological replicates were employed for qRT-PCR analysis.

### Construction of subcellular localization vectors and virus-induced gene silencing vectors

The full-length coding sequences (CDS) of the *NtFIP37B* spanning 300-500bp was used to design primers for constructing subcellular localization and virus-induced gene silencing (VIGS) vectors (Table S3). For the subcellular localization vector, the PCR-amplified insert was ligated into the homologous recombination vector PC1300s-*NtFIP37B*-GFP containing adapter primers for sequencing verification. The resulting construct was transformed into *Agrobacterium tumefaciens* GV3101, along with an empty vector control, and positive colonies were selected and grown in liquid cultures at 28°C until reaching an OD of 1. The bacteria were then resuspended in a mixture of MgCl_2_, acetosyringone (As), and 2-*N*-morpholinoethanesulfonic acid (MES) before being injected into *Nicotiana benthamiana* leaves in the dark for 3 h. The plants were then incubated in darkness for one day followed by one day in a greenhouse, after which their subcellular localization was assessed using confocal microscopy system (Nikon C2-ER, Japan). Similarly, the VIGS vector pYY13-*NtFIP37B* was constructed by homologous recombination and transformed into *A. tumefaciens* GV3101, along with TRV1, TRV2, and pTRV2-*PDS* controls. Positive colonies were selected and grown as above, and the bacteria were resuspended and injected into *N. benthamiana* leaves alongside negative controls (*A. tumefaciens* carrying pTRV2) and experimental groups (*A. tumefaciens* carrying pTRV2-*NtFIP37B*) as infecting agents. The bacteria carrying pTRV1 and pTRV2, pTRV1 and pTRV2-*PDS*, and pTRV1 and pTRV2-*NtFIP37B* were mixed in a 1:1 ratio and injected into the lower leaves of *N. benthamiana* using a sterile syringe. After one day of incubation in darkness, the plants were transferred to a greenhouse for 14 days. Once the bleaching phenotype was observed in the leaves inoculated with TRV2-*PDS*, the expression levels of newly grown leaves on the same site inoculated with TRV2-*NtFIP37B* were analyzed. Subsequently, silenced plants were subjected to drought treatment, and *Fv*/*Fm* ratios were calculated using IMAGING-PAM Chlorophyll Fluorescence System.

## Results

### Genome-wide identification and characterization of m^6^A regulatory genes in tobacco

**Fig. 1:**
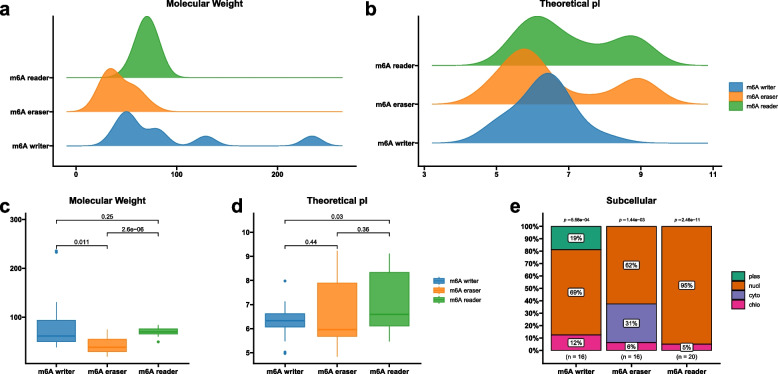
Distribution and statistic analysis for (**a** and **c**) molecular weight, (**b** and **d**) isoelectric point and (**e**) percentage of subcellular localization of tobacco m^6^A regulatory proteins. The *P* values in c and d were analyzed by ‘*t-test*’ method

After comprehensive screening of the tobacco genome, a total of 16 m^6^A writers, 16 m^6^A erasers and 20 m^6^A readers were finally identified (Table S4). All m^6^A regulatory genes were named according to their homologs of *Arabidopsis*, tomato and rice. Protein length and predicted molecular weight (MW) were significantly different between three types of m^6^A regulatory genes (Fig. 1a-d), especially for m^6^A writer members. The lengths of putative m^6^A writer proteins ranged from 341 (NtFIP37A/B) to 2,153 (NtVIR2) amino acid, and the MW of proteins ranged from 38.56 (NtFIP37A) to 235.81 (NtVIR2) kDa. Commonly, the length and MW of m^6^A writer proteins with the same domain were similar, except for NtMTB1/2 and NtMTB3/4. Although all m^6^A eraser proteins belonged to ALKBH family, their protein length and MW varied greatly, from the smallest NtALKBH6 (176 aa, 19.38 kDa) to the largest NtALKBH9B1 (668 aa, 75.09 kDa). In contrast, protein length and MW was similar among m^6^A reader proteins, with protein length (443 ~ 764 aa, mean = 636.95 aa) and MW (49.6 ~ 84.11 kDa, mean = 70.051 kDa). Similarly, theoretical isoelectric points also varied widely among m^6^A regulatory proteins. NtALKBH9B2 had the highest theoretical isoelectric point (9.23), while NtALKBH7A had the lowest one (4.84). In addition, subcellular localization prediction showed that most m^6^A regulatory proteins were located in the nucleus (40/52: 77%), and only several proteins were located in the cytoplasm (NtALKBH6, NtALKBH7A, NtALKBH8B and NtALKBH9B1/2), plasma membrane (NtMTC1 and NtVIR1/2) and chloroplast (NtMTA1/2, NtALKBH10 and NtYTHDF3D) (Fig. 1e).

### The phylogenetic classifications and structural features of m^6^A regulatory genes in tobacco

**Fig. 2:**
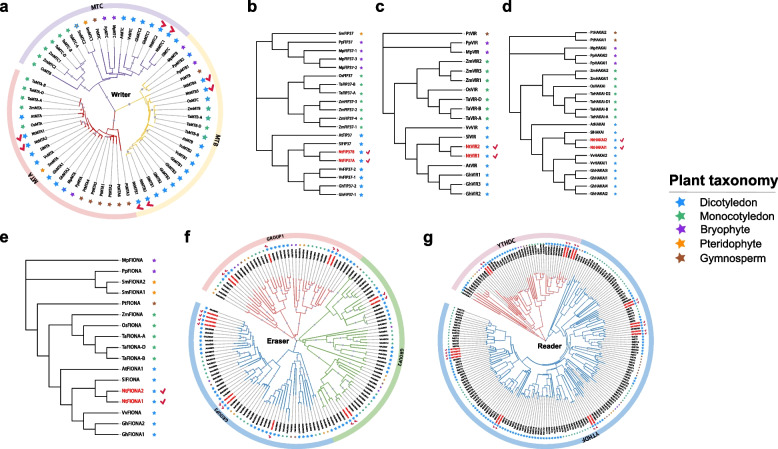
Phylogenetic analysis of m^6^A regulatory genes from five dicotyledons, three monocotyledons, one pteridophyte, two mosses, and one gymnosperm. **a** Phylogenetic tree of MT-A70 genes. **b** Phylogenetic tree of FIP37 genes. **c** Phylogenetic tree of VIR genes. **d** Phylogenetic tree of HAKAI genes. **e** Phylogenetic tree of FIONA genes. **f** Phylogenetic tree of m^6^A eraser genes. **g** Phylogenetic tree of m^6^A reader genes. Stars of various hues signify distinct categories of species in accordance with the legend. The tobacco m^6^A regulatory genes are highlighted in bold red letters and marked with a check symbol

To gain an insight into the evolutionary history of m^6^A regulatory genes in tobacco, the protein sequences of m^6^A writer, eraser and reader from five dicotyledons, three monocotyledons, one gymnosperm, one pteridophyte and two mosses were used for phylogenetic analysis. The results showed that m^6^A writers could be naturally grouped into five categories, namely MT, FIP37, VIR, HAKAI, and FIONA (Fig. 2a-e). The MT group was further divided into three subgroups: MTA, MTB and MTC, which belonged to their evolutionary clades. Among all the plants used for phylogenetic analysis, MTB, HAKAI and VIR subfamilies were absent from the pteridophyte and *S. moellendorffii*, whereas other subfamilies were present in all plants, suggesting different evolutionary roles of m^6^A writers among different plants. Most diploid plants contained single copy of FIONA, while polyploid plants usually had 2 to 3 paralogs of FIONA protein. The phylogenetic tree of ALKBH family of m^6^A eraser was clustered into three groups, with a relatively uniform distribution in all plants (Fig. 2f; Table S5). Phylogenetic analysis showed that YTH proteins of m^6^A reader were grouped into two main categories, namely YTHDF and YTHDC (Fig. 2g). Compared with m^6^A writers and eraser, the number of genes encoding YTH domain proteins was much greater, especially for YTHDF subgroups. Interestingly, m^6^A regulatory proteins from the same plant clade tended to cluster together. Most m^6^A proteins from mosses, pteridophytes and gymnosperms were located in a same branch, whereas dicotyledons and monocotyledons tended to be in other branches. In addition, as a typical allotetraploid plant, tobacco had more m^6^A regulatory genes than diploid plants such as *Arabidopsis*, grape and rice (Table S5). Similar results could be observed in hexaploid wheat and tetraploid cotton, suggesting gene family expansion for polyploidy plants [66].

**Fig. 3:**
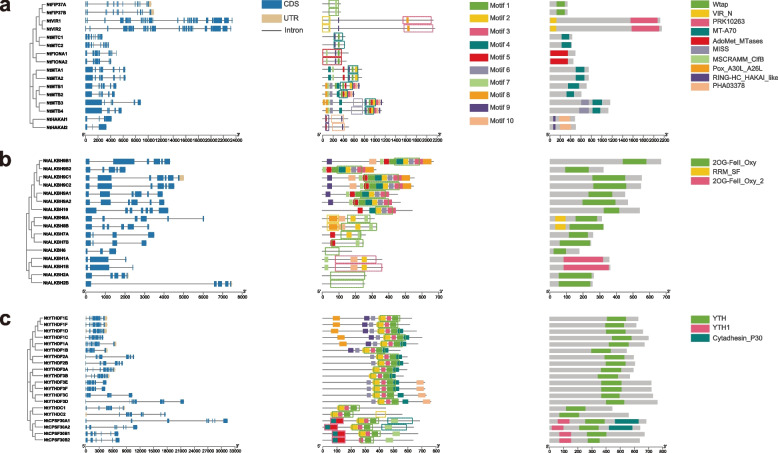
Phylogenetic relationships, gene structures, conserved motifs, and functional domains of m^6^A regulatory genes in tobacco. **a** Structure analysis of m^6^A writer genes. **b** Structure analysis of m^6^A eraser genes. **c** Structure analysis of m^6^A reader genes. The squares in the motif represent the positions of conserved domains

To better understand the evolution of m^6^A regulatory genes in tobacco, TBtools and MEME Suite were used to explore their sequence characteristics, conserved domains and consensus motifs (Fig. 3). For m^6^A writer, the genes from the same branch had similar exon–intron structures and conserved domains, except that *NtMTB3* had one more exon than *NtMTB4*. Interestingly, NtVIRs had a super long structure with 27 to 28 exons, which was also found in other plants such as tea [48] and poplar [67]. MT-A70 domain was found in all tobacco MT families, and NtMTB proteins also contained a MAPK-interacting and spindle-stabilizing protein-like protein domain (MISS). Both NtVIR proteins contained a conserved VIR_N domain and a PRK10263 domain, and all NtHAKAI proteins contained two conserved domains, RINGHC_HAKAI_like and PHA03378. Two homologs of *Arabidopsis FIONA1* genes in tobacco contained the AdoMet_MTase domain. Compared with m^6^A writer members, more structural variations were found in m^6^A eraser genes. *NtALKBH9B1* had one larger exon than *NtALKBH9B2*. The exon number and intron length of *NtALKBH8A* were significantly different from *NtALKBH8B*. In addition, *NtALKBH2B* had one bigger intron. All m^6^A eraser members contained the 20G-FeeII_Oxy domain, and *NtALKBH8* subfamily also had another conserved domain of RRM_SF. Structural variations could also be observed in m^6^A reader genes. *NtYTHDF3D* had longer introns than *NtYTHDF3C*, and *NtYTHDC1* lacked two exons compared with *NtYTHDC2*. In addition, *NtCPSF30A1* had one more exon and longer intron than *NtCPSF30A2*. The YTH conserved domain could be found in all tobacco m^6^A reader proteins, while the CPSF30 subfamily also had another YTH1 conserved domain.

Motifs analysis revealed that the genes from the same branch had similar motif composition. For m^6^A writer, the number of motifs varied from 2 to 10, and the NtMTB subfamily contained all 10 motifs. Interestingly, two unknown motif (7 and 9) could be found in most m^6^A writer members (Fig. 3a; Table S6). Among ALKBH families, no conserved motif was identified in *NtALKBH6* and *NtALKBH2A/B*, which also had distinguished structures. Most m^6^A eraser genes had a similar motif composition for each branch. Two YTH domain-containing protein motifs (1 and 3) were existed in all m^6^A reader proteins. In general, most of the m^6^A regulatory proteins in same branch exhibited similar motif distribution and exon–intron structure.

### The chromosomal distributions, gene duplication events and collinearity analysis of m^6^A regulatory genes in tobacco

Among the identified m^6^A regulatory genes, 21 were randomly distributed on 10 chromosomes, and the remaining ones were located on the unanchored scaffolds (Table S4). Chromosome 17 contained the most m^6^A regulatory genes (4), two m^6^A eraser and two m^6^A reader genes, respectively. There was only one gene on chromosomes 1, 2 and 14.

Considering that gene duplication is an important mechanism for gene family expansion, we utilized the DupGen_finder softwaree [52] to identify different duplication events of m6A regulatory genes in tobacco. The analysis revealed a total of 14 duplication events, including 10 instances of dispersed duplication (DSD), 3 cases of tandem duplication (TRD), and 1 occurrence of proximal duplication (PD), involving a set of 18 genes responsible for m6A regulation. However, we did not detect any instances of whole-genome duplication (WGD) or transposed duplication (TRD) (Table S7). Notably, although there were several gene pairs with high similarity in amino acid sequence, such as NtYTHDF1E and NtYTHDF1F (with a similarity of 96.019%), we were unable to determine their modes of duplication due to the absence of chromosomal location. Interestingly, two pairs of TRD genes (*NtALKBH9B1*-*NtALKBH9B2* and *NtCPSF30A1*-*NtCPSF30A2*) were anchored to chromosome 17, while another pair of TRD genes were located on the same scaffold. The intricate network of DSD events might play an important role in the expansion of MT-A70, ALKBH and YTH gene families. Besides, DSD was the main event (4/6: 66.7%) for tobacco m^6^A reader genes. Interestingly, several genes might be involved in two or three rounds of dispersed duplication events, including *NtMTA1*, *NtCPSF30A1* and *NtYTHDF3E*.

To explore the evolutionary trajectory of duplicated gene pairs in m^6^A regulatory genes of tobacco, we calculated the ratio of non-synonymous (*Ka*) and synonymous (*Ks*) substitution rates (Table S7). Typically, *Ka*/*Ks* ratios below 1, equal to 1, and above 1 indicated purifying selection, neutral selection, and positive selection, respectively. In this study, all analyzed duplication events yielded *Ka*/*Ks* ratios were less than 1, indicating the predominance of strong purifying selection on these genes throughout their evolutionary history. This suggested that functional differentiation following gene duplication had been limited by the action of purifying selection. Based on *Ks* values, we estimated divergence times of m^6^A duplicated gene pairs in tobacco. The *Ks* values of m^6^A duplicated gene pairs ranged from 0.047 to 2.609, with estimated divergence times ranging from 1.567 Mya to 86.975 Mya. Duplicated gene pairs with earlier divergence times (such as *NtMTA1* and *NtMTC1*, *NtYTHDF1A* and *NtYTHDF3E*) might be associated with ancient duplication events, while those with later divergence times (such as *NtCPSF30A1* and *NtCPSF30A2*, *NtALKBH9B1* and *NtALKBH9B2*) might be likely due to more recent dispersed or tandem events. These findings shed light on the evolutionary history of m^6^A duplicated genes in tobacco.

**Fig. 4:**
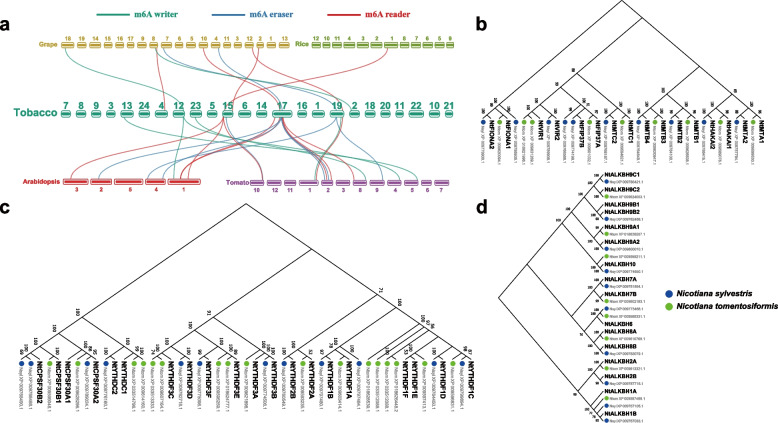
Collinearity analysis of tobacco m^6^A regulatory genes and inference of their diploid origins based on evolutionary relationships. **a** Micro-collinearity relationship between m^6^A regulatory genes in tobacco and those in *Arabidopsis*, grape, tomato, and rice. The colors represented by each line are indicated in the legend. **b**-**d** Phylogenetic analysis of tobacco m6A writer (**b**), reader (**c**), and eraser (**d**) and their diploid progenitors *N. sylvestris* and *N. tomentosiformis*

To further explore the evolutionary patterns of m^6^A regulatory genes between different plants, four representative species were selected for comparative collinearity analysis with tobacco. Among the twenty m^6^A regulatory genes located on chromosomes in tobacco, 13 were collinear with tomato, followed by *Arabidopsis* (8), grape (8) and rice (1) (Fig. 4a). Obviously, the number of m^6^A regulatory genes with collinearity between monocotyledons and dicotyledons varied widely, suggesting that the expansion of m^6^A regulatory genes might occur after dicotyledonous differentiation. In addition, one eraser gene (*NtALKBH9B1*) and two reader genes (*NtYTHDF3E* and *NtCPSF30A1*) had more than one pair of collinear genes.

As a typical allotetraploid plant, tobacco was formed from the maternal donor *N. sylvestris* and the male donor *N. tomentosiformis* about 0.2 million years ago [40]. To explore the parental origin of m^6^A regulatory genes in tobacco, m^6^A regulatory genes were also identified in two genome donors of tobacco. To investigate the parental origin of m^6^A regulatory genes in tobacco, a phylogenetic tree was conducted between tobacco and its ancestor donors (Fig. 4b-d; Table S4). Phylogenetic analysis showed that the m^6^A writer genes in tobacco were equally derived from two diploid donors, respectively. Compared with its donors, some FIP37 and VIR members were lost during the evolution of nearly 0.2 million years, and no expansion was found for these two families. For the ALKBH family, *NtALKBH1A* and *NtALKBH6* could not assign their origins according to the phylogenetic tree, suggesting these two genes might have experienced differentiation during evolution. Our previous analysis revealed that one TRD event have occurred in *NtALKBH9B* during evolution, which might be the major reason for *NtALKBH9B1* and *NtALKBH9B2* were located in the same branch with *N. sylvestris*. Similarly, *NtYTHDC1C*-*NtYTHDC1D* and *NtCPSF30A1*-*NtCPSF30A2* might also expand during evolution. Similar results could also be observed for *NtYTHDF1E*-*NtYTHDF1F* (*N. tomentosiformis* origin) and *NtYTHDF3A*-*NtYTHDF3B* (*N. sylvestris* origin). However, their duplication patterns could not be analyzed because they could not be located on chromosomes. We speculated that these two pairs of genes might also have experienced post-speculation TRD events during evolution.

### Analysis of *cis*-acting regulatory elements (CREs) of m^6^A regulatory genes in tobacco

**Fig. 5:**
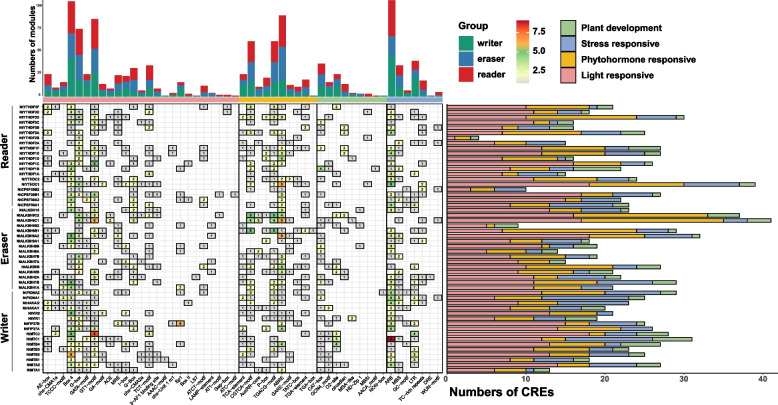
The distribution of *cis*-acting elements in the promoter regions of m^6^A regulatory genes. The bar charts situated above and the right of the heatmap depict the statistical results for horizontal and vertical comparisons, respectively. The color for different categories is described in the legend

The *cis*-acting elements (CREs) in the promoter region of genes played an important role in determining the spatiotemporal specific expression of protein-coding genes [68]. To better understand the transcriptional regulation and potential biological functions of tobacco m^6^A regulatory genes, the CREs in the promoter regions (2,000 bp upstream of the transcription start site) of m^6^A regulatory genes were predicted using the PlantCare database. These identified CREs can be further divided into four types (Fig. 5; Table S8), including light-responsive (46%), phytohormone-responsive (27%), stress-responsive (18%) and development-related elements (9%). All m^6^A regulatory genes contained a large number of light-responsive elements, indicating that they may be regulated by light signaling. Abscisic acid (ABA) and methyl jasmonic acid (JA) responsive elements accounted for the majority of phytohormone-responsive elements, including 91 and 124, respectively. These large number of phytohormone-responsive elements indicated that hormone signals may activate or inhibit the expression level of m^6^A regulatory genes. Among the CREs associated with plant development, we observed enrichment of CAT-box, GCN4_motif, and O2-site in the promoter regions of m^6^A regulatory genes in tobacco. These CREs were respectively associated with meristem expression, endosperm expression, and metabolism regulation. In addition, we found that ARE elements, involved in anaerobic induction, were present in the promoter regions of the majority of m^6^A regulatory genes. MBS (related to drought response) and LTR (related to cold response) elements were also highly enriched (Fig. 5). These findings indicated tobacco m^6^A regulatory genes might respond to a variety of external stresses and internal metabolic types.

### Regulation networks of m^6^A regulatory genes in tobacco

**Fig. 6:**
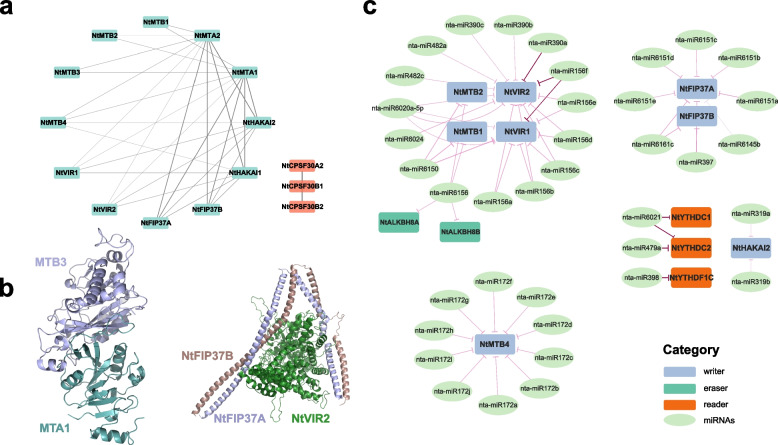
Potential m^6^A regulatory network in tobacco. **a** Protein–protein interaction network of m^6^A regulatory proteins in tobacco. Edge thickness represents the combined score from STRING. **b** Protein docking results of NtMTA1-NtMTB3 (left) and NtVIR2-NtFIP37A-NtFIP37B (right) complexes. **c** Interaction between m^6^A regulatory genes and miRNAs. Edge thickness represents the negative UPE value, indicating the likelihood of miRNA-mediated interaction and cleavage of target mRNAs. Edge color intensity reflects the number of mismatches, with darker colors indicating fewer mismatches

Considering that the m^6^A modification was performed by a multi-protein complex consisting of a catalytic core (MTA and MTB) and a series of auxiliary subunits acting together, we constructed a potential protein–protein interaction network of tobacco m^6^A regulatory genes by STRING database (Fig. 6a; Table S9). Among the tobacco m^6^A writer members, except for the NtMTC and NtFIONA subfamilies, all the other 12 members showed inter-interactions, especially the NtMTA and NtHAKAI subfamilies, suggesting that they may participate in m^6^A modification by forming protein complexes. However, no interaction between m^6^A eraser members was found. *NtCPSF30B1* could also interact with two other NtCPSF30 subfamily members (*NtCPSF30A2* and *NtCPSF30B2*), which may be related to their special roles in recognizing m^6^A modification (Fig. 6a). Considering that mRNA m^6^A modification was catalyzed by the m^6^A methyltransferase complex [9], we predicted 3D structural models of tobacco m^6^A writer proteins using a homology modeling approach, and performed protein–protein docking of different combinations (Fig. 6b; Table S10). The results indicated that the docking model of NtMTA-NtMTB exhibited a higher docking confidence score ranging from 0.8861 to 0.9999, suggesting a significant likelihood of protein complex formation. Among them, NtMTA1-NtMTB3 protein complex model with the highest docking confidence score (0.9999) exhibited a similar conformation to the human METTL3-METTL4 complex (PDB accession: 5L6D) [69] (Fig. 6b). Surprisingly, NtFIP37A and NtFIP37B also exhibited a high docking confidence score (0.9998). Studies have shown that two WTAP (ortholog of FIP37) molecules formed an asymmetric homodimer, acting as a "bridge" connecting VIRMA with the METTL3-METTL14 complex [9]. By docking NtVIR2 with the NtFIP37A-NtFIP37B protein complex, we found that the resulting ternary complex model exhibited a structure similar to the human m6A-METTL associated complex (WTAP and VIRMA complex, PDB accession: 7VF5) [70] (Fig. 6b). Consequently, we proposed that FIP37 proteins in plants might possess similar functions.

Subsequently, potential miRNA binding sites of m^6^A regulatory genes were also investigated by psRNATarget [59]. After filtering interactions with an Expectation penalty score below 5, a total of 38 miRNAs from 16 families were identified as potential regulators targeting m^6^A regulatory genes including 8 writers, 3 readers, and 2 erasers (Fig. 6c; Table S9). Notably, it has been observed that distinct miRNA families often exhibited specific target genes. For instance, *NtMTB4*was potentially regulated by ten miRNAs, all belonging to the nta-miR172 family. Furthermore, six miRNAs from the nta-miR156 family could target the *NtVIRs* genes (Fig. 6c). Interestingly, compared to the m^6^A readers (*n* = 3) and m^6^A erasers (*n* = 1), tobacco m^6^A writers (*n* = 35) appeared to be subjected to greater miRNAs regulation. These m^6^A regulatory genes were predominantly clustered within the MTB, VIR, and FIP37 families. However, no miRNAs regulating the MTA and MTC subfamilies in tobacco were identified in our analysis. Considering the context-dependent activation of miRNA-mediated regulation, further investigations were needed to unravel the intricate interplay between m^6^A regulatory genes and miRNAs in plants.

### Tissue expression analysis of m^6^A regulatory genes in tobacco

**Fig. 7:**
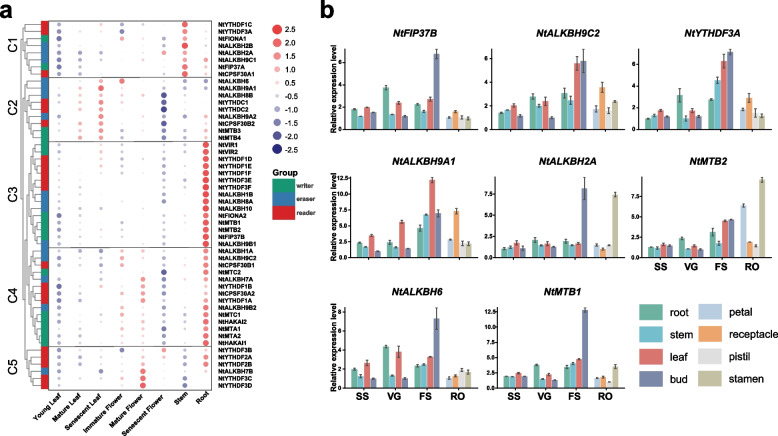
Expression profiles of tobacco m^6^A regulatory genes in different tissues. **a** Heatmap of the expression in different tissues, scaled by rows. **b** qRT-PCR analysis of nine selected m^6^A regulatory genes in different tissues at various developmental stages. Data are presented as mean ± SD

To explore the expression patterns of m^6^A regulatory genes in different tissues of tobacco, the expression profiles of eight representative tissues were explored (Fig. 7a, Table S11). As depicted in Fig. 7a, the expression patterns of m^6^A regulatory genes could be classified into five distinct clusters (C1-C5). Notably, clusters C1-C4 comprised approximately three types of m^6^A regulatory genes, suggesting potential co-regulation among them. However, in cluster C5, the majority of genes belonged to the m^6^A reader category, with only one gene from m^6^A eraser (*NtALKBH7B*). Genes in cluster C1 exhibited specific expression in stems, and relatively low in leaves. In cluster C2, gene expression gradually increased during leaf development, such as *NtALKBH9A1*, *NtYTHDC1*, and *NtMTB3*, implying their roles in leaf development. Genes in clusters C3 and C4 displayed high expression levels in roots and showed minimal or no expression in senescent flowers, indicating functional divergence of m^6^A regulatory genes during tobacco tissue development. Importantly, compared to immature flowers, the majority of genes in clusters C4 and C5 exhibited significant up-regulation in mature flowers, followed by a notable decrease in senescent flowers. This trend was particularly evident for certain m^6^A eraser and m^6^A reader members, such as *NtYTHDF3C*, *NtYTHDF3D*, *NtALKBH7A*, and *NtALKBH7B*, highlighting their dynamic regulation during tobacco flower organ development. Additionally, we observed a strong positive correlation in the expression of numerous genes across different tissues (Fig. S1a), suggesting a cooperative impact among m^6^A genes in tobacco. Furthermore, we randomly selected eight tobacco m^6^A regulatory genes with specific expression patterns in different tissues for further qRT-PCR analysis at various developmental stages, including *NtFIP37B*, *NtMTB1*, *NtMTB2*, *NtALKBH2A*, *NtALKBH9A1*, *NtALKBH9C2*, *NtALKBH6*, and *NtYTHDF3A*. Most of the qRT-PCR results were consistent with the RNA-seq data (Fig. 7b). Among the selected tobacco m^6^A regulatory genes, FS (Flowering stage) exhibited significantly higher expression levels in all tissues, particularly in buds, indicating the important role of m^6^A modification during reproductive growth stages. Additionally, individual tobacco m^6^A regulatory gene showed relatively high expression levels in specific floral organs, such as *NtALKBH9A1* in the receptacle and *NtALKBH2A* and *NtMTB2* in the stamen, suggesting their potentially significant roles in floral organ development.

### Expression patterns of m^6^A regulatory genes in tobacco under various abiotic and biotic stresses

**Fig. 8:**
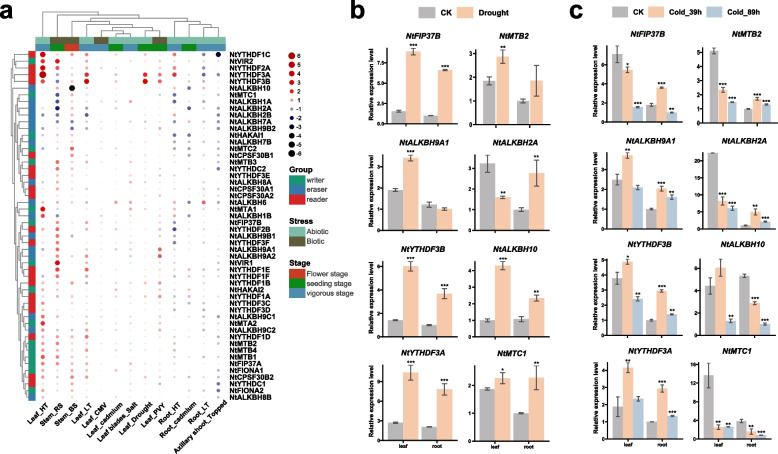
Expression profiles of tobacco m^6^A regulatory genes under various stress treatments. **a** Heatmap of the expression under high temperature (HT), low temperature (LT), *R. solanacearum* (RS), black shank (BS), Cucumber Mosaic Virus (CMV), cadmium, salt, drought, Potato Virus Y (PVY) and topping treatment. The expression change is indicated by the ratio of *TPM* value between the treatment and control (CK). **b**, **c** qRT-PCR analysis of eight selected m^6^A regulatory genes under drought (**b**) and cold (**c**) treatment. The samples were collected from 28d-old tobacco (vigorous stage). Data are presented as mean ± SD, with one, two, and three asterisks denoting statistical significance at *p* < 0.05, *p* < 0.01, and *p* < 0.001, respectively

To unveil the possible function of m^6^A regulatory genes in response to different stresses, the expression pattern of m^6^A regulatory gene under various stresses was studied (Fig. 8; Table S12). Our analysis revealed that m^6^A regulatory genes exhibited distinct response patterns under different stresses. The majority of m^6^A regulatory genes did not show significant changes relative to the control when tobacco was subjected to CMV or salt stress. Notably, the response pattern of m^6^A regulatory genes differed between leaf and root tissues under similar stresses such as high temperature, low temperature, and cadmium. For instance, while *NtYTHDF3A* and *NtYTHDF3B* were significantly up-regulated in leaves at low temperature, they were down-regulated in roots. Similarly, *NtALKBH10*, *NtALKBH2A*, *NtHAKAI1* and *NtALKBH6* displayed a significant decrease in cadmium-treated tobacco root tissue but exhibited no change or up-regulation in leaves, implying tissue specificity of m^6^A regulatory genes in response to different stress. Several m^6^A reader genes including *NtYTHDF1C*, *NtYTHDF2A* and *NtYTHDF3A*, especially *NtYTHDF3A*, were significantly up-regulated in response to high temperature, hinting their potential roles in high temperature stress. The expression of both genes, *NtYTHDF3A* and *NtYTHDF3B*, were significantly increased under drought stress, which were also highly expressed in leaves under high/low temperature. Interesting, after topping treatment, the expression of almost all tobacco m^6^A regulatory genes were reduced relative to the control. Comparing with black shank (BS) and Potato Virus Y (PVY), the *Ralstonia solanacearum* (RS) treatment resulted in the relative higher up-regulation of multiple genes, such as *NtVIR1*, *NtVIR2*, *NtYTHDF2A*, and *NtYTHDF1E*. Apart from Cucumber mosaic virus (CMV), the expression patterns of tobacco m^6^A regulatory genes varied greatly in response to biotic stresses including RS, BS and PVY, with only one gene, *NtALKBH9A2*, being significantly up-regulated in all three biotic stresses.

Additionally, a substantial positive correlation was identified in the expression of multiple tobacco m^6^A regulatory genes under various stress treatment (Fig. S1b). We focused on eight genes that showed significant responses to drought and cold stress, and further investigated their expression patterns using qRT-PCR experiments (Fig. 8b, c). Under drought stress, the expression levels of six genes (*NtFIP37B*, *NtYTHDF3B*, *NtALKBH10*, *NtYTHDF3A*, and *NtMTC1*) were significantly up-regulated in both roots and leaves. Notably, *NtFIP37B* exhibited a more than five-fold increase in leaves. Under drought stress, *NtALKBH9A1* and *NtALKBH2A* showed opposite expression patterns in leaves and roots, indicating tissue-specific responses to drought stress (Fig. 8b). *NtMTC1*, which was significantly up-regulated under drought stress, showed a significant down-regulation in expression under cold stress (Fig. 8c), suggesting different response patterns of m^6^A modification under different stress conditions. Additionally, we observed different dynamic expression patterns between root and leaf of multiple genes under cold treatment. From 36 to 89 h of cold stress, *NtFIP37B*, *NtALKBH9A1*, *NtYTHDF3B*, *NtALKBH10*, and *NtYTHDF3A* exhibited a pattern of initially increased expression followed by decreased expression in leaves or roots, indicating dynamic response patterns to cold stress. Overall, the expression patterns of m^6^A regulatory genes in tobacco under different stress conditions suggested that they might play critical biological roles in various stress responses.

### Silencing of *NtFIP7B* reduced drought tolerance of tobacco

**Fig. 9:**
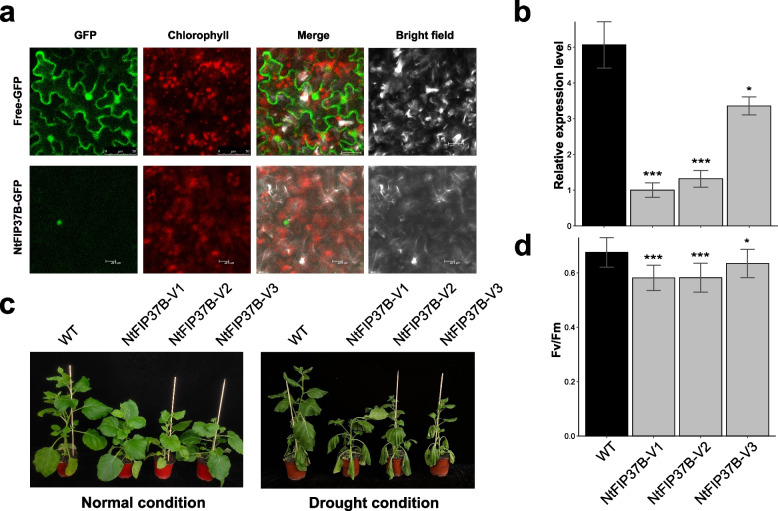
Subcellular localization of *NtFIP37B* and drought tolerance assessment in *NtFIP37B*-silenced tobacco plants. **a** Subcellular localization of *NtFIP37B*. The scale bars represent 20 μM. **b** Relative expression of *NtFIP7B* detected by qRT-PCR after drought treatment. **c** Phenotype and (**d**) *Fv*/*Fm* ratios of *NtFIP37B*-silenced tobacco plants before and after 5 days of drought treatment. Data are presented as mean ± SD, with one, two, and three asterisks denoting statistical significance at *p* < 0.05, *p* < 0.01, and *p* < 0.001, respectively

We have discovered that one tobacco m^6^A writer genes, named *NtFIP37B*, exhibited significant up-regulation under drought stress (Fig. 8b). To further explore the influence of m^6^A modification on tobacco's drought tolerance, we employed a virus-induced gene silencing system (VIGS) to construct silenced plants for *NtFIP37B*. Subsequent subcellular localization studies indicated that *NtFIP37B* were situated within the nucleus (Fig. 9a), which was consistent with predicted results (Table S4) as well as findings in other species [1]. Compared with wild-type plants, positively silenced plants showed significantly decreased transcript abundances of *NtFIP37B* (Fig. 9b). Under normal growth conditions, significant differences in plant height were observed between positively-silenced *NtFIP37B* plants (*NtFIP37B*-V1, *NtFIP37B*-V2, *NtFIP37B*-V3) and wild-type plants (Fig. 9c), indicating that this gene may have a positive regulatory role in tobacco growth. After five days of drought treatment, *NtFIP37B*-silenced plants exhibited more severe wilting (Fig. 9c) and a significant decrease in *Fv*/*Fm* ratio (Fig. 9d) compared to the wild type, indicating that their photosynthesis was severely affected. These results suggested that silencing of *NtFIP37B* inhibited growth of tobacco, and enhanced drought sensitivity in tobacco plants.

## Discussion

RNA *N*^6^-methyladenosine plays an important regulatory role in plant growth and development. Three types of m^6^A regulatory genes dynamically control the methylation levels of target transcripts involved in development and stress responses. Based on evidence from *Arabidopsis*, tomato, and rice, 52 candidate m^6^A regulatory genes were identified in tobacco, including 16 writers, 16 erasers, and 20 readers (Table S4). Although it has been speculated that a HAKAI-interacting zinc finger protein 2 (HIZ2) in *Arabidopsis* shares similarities with ZC3H13, its specific biological process in m^6^A multi-protein complex remains unclear [20]. Furthermore, due to the absence of obvious conserved domains in the amino acid sequence of HIZ2, it is not included in the scope of this study. The number of m^6^A regulatory genes in tobacco (52) exceeded that of several species, including *Arabidopsis* (33), tomato (25), grape (40), rice (33), *S. moellendorffii* (22), *M. polymorpha* (16), *P. patens* (18), and Chinese pine (36) (Table S5). However, it is lower than maize (55), common wheat (85), and upland cotton (75). Despite having the largest genome size (25.4 Gb), Chinese pine exhibits a remarkably low number of m^6^A regulatory genes, with only 36 members, which is significantly lower compared to species such as common wheat and upland cotton. Notably, the species with a higher abundance of m^6^A regulatory genes have experienced polyploidization events. Common wheat (2n = 6x = 42), upland cotton (2n = 4x = 52), and tobacco (2n = 4x = 48) are all the result of hybridization between two or multiple diploid donor species [71–74], leading to their m^6^A regulatory gene count being two or three times that of diploid species like *Arabidopsis*. Likewise, maize, a segmental allopolyploid [75], also possesses a higher number of m^6^A regulatory genes. Additionally, a total of 14 duplication events were identified, involving 18 m^6^A regulatory genes in tobacco. Dispersed duplication was the most frequent event (10), followed by tandem duplication (3) and proximal duplication (1) (Table S7). *Ka* and *Ks* analysis revealed that duplicated gene pairs have undergone purifying selection, consistent with findings in studies of tea plants and tomatoes [48, 49]. Estimated divergence times based on *Ks* values indicated that the duplication of m^6^A regulatory genes in tobacco predated the formation of the common tobacco species (~ 0.2 Mya) [40]. Phylogenetic analysis showed distinct subfamilies within m^6^A regulatory genes, with bryophytes, pteridophytes, and gymnosperms clustering separately from monocots and dicots [76] (Fig. 2). The preferential expansion of m^6^A regulatory genes in monocots and dicots may be attributed to their expansion events following divergence. In summary, we proposed that the differences in the number of m^6^A regulatory genes among species were primarily attributed to polyploidy, whole genome duplication (WGD), and gene duplication events, and the duplicated m^6^A regulatory genes in tobacco underwent differentiation in its two-progenitor diploid donor and no duplication events occurred after species formation (Table S7).

Previous studies have shown that m^6^A modification determined mRNA fate via multiple aspects of mRNA metabolism, impacting various aspects such as alternative splicing, alternative polyadenylation, folding, translation, localization, transport, and decay, thereby regulating plant growth, development, and stress responses [1]. In tobacco, the m^6^A writer proteins exhibited strong interactions within a protein interaction network apart from the *FIONA1* and *MTC*, with NtMTAs and NtFIP37s showing the most interactions, highlighting their importance as the catalytic core and auxiliary subunit, respectively. The prediction results indicated that there were no interactions among eraser proteins in tobacco, while the three reader proteins including *NtCPSF30A2*, *NtCPSF30B1*, and *NtCPSF30B2* might have interactions, which required further research for validation (Fig. 6a). Using homology modeling and protein docking, the 3D structures of tobacco m^6^A regulatory genes were predicted, confirming the high likelihood of NtMTAs and NtMTBs forming the catalytic core for m^6^A methylation. Another important component of the MTC, NtVIR2-NtFIP37A-NtFIP37B, also exhibited a structure similar to that found in humans (Fig. 6b; Table S10). Moreover, similar expression pattern were observed for m^6^A writer genes under different stress treatment, suggesting their coordinated role in defense responses (Fig. S1). Additionally, potential miRNA binding sites were identified in tobacco m^6^A regulatory genes, indicating a possible role of miRNAs in regulating m^6^A methylation (Fig. 6; Table S9). Interestingly, compared to the m^6^A readers (*n* = 3) and m^6^A erasers (*n* = 1), m^6^A writers (*n* = 35) appeared to be subjected to greater miRNAs regulation. In other words, m^6^A writers in tobacco might prefer to be regulated by miRNAs (Fig. 6). Moreover, among all m^6^A writers, only MTB, VIR, and FIP37 families might targeted by miRNAs. Therefore, more future works were needed to explore the mutual interactions between m^6^A writer genes and miRNAs.

In order to explore potential functions for m^6^A regulatory genes in tobacco, the promoter regions were analyzed, and a significant enrichment of CREs associated with light response, hormone response, plant growth and development, and stress response were observed (Fig. 5; Table S8). Similar findings were also found in other plant species such as tea [48], tomato [49], and poplar [67]. The promoter regions of m^6^A regulatory genes in tobacco encompassed a substantial proportion (46%) of light-responsive CREs. In *Arabidopsis*, the m^6^A methyltransferase MTA [77] and FIONA1 [78] could indirectly influence the stability and translation efficiency of mRNA of biological clock genes through the blue light-induced photoreceptor CRY2, which played a crucial role in maintaining chlorophyll homeostasis in plants under light conditions. Transcriptome analysis revealed high expression levels of m^6^A regulatory genes in tobacco roots (Fig. 7a). Previous studies had shown that *CPSF30L* in *Arabidopsis* could regulate APA by binding to m^6^A-modified transcripts involved in nitrate signaling, such as *NRT1.1* and *WRKY1* [79]. Considering the crucial role of roots in nitrogen absorption, it was plausible to hypothesize that NtCPSF30s in tobacco might function through similar mechanisms. Additionally, qRT-PCR results showed that many tobacco m^6^A regulatory genes such as *NtFIP37B*, *NtALKBH9C2*, and *NtYTHDF3A* were highly expressed in multiple tissues and organs during the flowering period. These highly expressed m^6^A regulatory genes might play crucial roles in reproductive development, as previous studies have associated m^6^A modification with reproductive cell development [80] and fruit ripening [29, 81]. The promoter regions of tobacco m^6^A regulatory genes contained stress-responsive elements associated with diverse biotic and abiotic stresses (Fig. 5). Analysis of m^6^A gene expression patterns under different stress conditions revealed various responsive patterns. For instance, under high temperature stress, *NtVIR2* showed significant up-regulation while *NtVIR1* remained stable, indicating functional redundancy. Moreover, genes like *NtALKBH10* and *NtVIR1* exhibited robust responses to specific biotic stresses (RS and BS) but showed insensitivity to CMV or PVY infection (Fig. 8a). Although barley and soybean exhibited increased global m^6^A modification levels in response to cadmium stress [82, 83], we did not observe significant up-regulation of m^6^A writer genes in tobacco (Fig. 8a), possibly due to the different experimental conditions. Differential response times to drought treatment were observed among various m^6^A regulatory genes. For instance, genes like *NtFIP37B* and *NtALKBH10* did not show significant changes in expression levels after 4 h of drought treatment (Fig. 8a). However, when tobacco plants were exposed to drought treatment for 7 days, these genes exhibited significant upregulation (Fig. 8b). In summary, the expression patterns of tobacco m^6^A regulatory genes under growth, development, and stress conditions were diverse, implying their functional diversity. Further investigations were needed to elucidate the detailed mechanisms of candidate m^6^A regulatory genes in tobacco.

## Conclusions

This study presents the first comprehensive and systematic investigation of m^6^A regulatory genes that may be involved in *N*^*6*^-methyladenosine modification in tobacco. A total of 52 m^6^A regulatory genes were identified in tobacco, categorized into three types: m^6^A writer (16), m^6^A eraser (16), and m^6^A reader (20). Subsequently, we analyzed the features of these genes in terms of gene structure, conserved domains, and motifs. Through systematic evolutionary analysis, collinearity analysis, and identification of duplicate genes, we discovered that polyploidization and segmental duplication were the main drivers for the expansion of tobacco m^6^A regulatory genes. Functional analysis of tobacco m^6^A regulatory genes in *cis*-elements, interaction networks, and expression patterns demonstrated their crucial roles in tobacco growth, development, and stress responses. Notably, *NtFIP37B*-silenced tobacco plants exhibited significantly reduced drought tolerance. This study provides a reference framework for exploring the functional diversity of m^6^A regulatory genes in tobacco growth, development, and stress responses at the epigenetic level. These findings are of significant importance for enhancing tobacco plant resistance to stress and require further elucidation in future work.

### Supplementary Information

**Supplementary Material 1. **

**Supplementary Material 2. **

## Data Availability

All data generated or analyzed in this study are included in the materials and methods section of this article.
