# Supplementary material for: Genome-wide identification of the N6-methyladenosine regulatory genes reveals NtFIP37B increases drought resistance of tobacco (Nicotiana tabacum L.)
Source: BMC Plant Biol. 2024 Feb 26;24:134. doi: 10.1186/s12870-024-04813-2 (PMC10895791; doi:10.1186/s12870-024-04813-2)
Supplement: Supplementary file 2 — Supplementary Material 2. [file 12870_2024_4813_MOESM2_ESM.docx]

**Fig. S1** The expression correlation of m^6^A regulatory genes in tobacco. (A) Heatmap of expression correlation in different tissues. (B) Heatmap of expression correlation under different stresses. The correlation was calculated using *Pearson's* method, and non-significant correlations were displayed as blank spaces in the heatmap.
